# Computational Method‐Based Optimization of Carbon Nanotube Thin‐Film Immunosensor for Rapid Detection of SARS‐CoV‐2 Virus

**DOI:** 10.1002/smsc.202100111

**Published:** 2021-11-16

**Authors:** Su Yeong Kim, Jeong-Chan Lee, Giwan Seo, Jun Hee Woo, Minho Lee, Jaewook Nam, Joo Yong Sim, Hyung-Ryong Kim, Edmond Changkyun Park, Steve Park

**Affiliations:** ^1^ Organic and nano electronics laboratory KI for Health Science and Technology Department of Materials Science and Engineering Korea Advanced Institute of Science and Technology (KAIST) Daejeon 34141 Republic of Korea; ^2^ Research Center for Bioconvergence Analysis Korea Basic Science Institute Cheongju 28119 Republic of Korea; ^3^ Center for Convergent Research of Emerging Virus Infection Korea Research Institute of Chemical Technology Daejeon 34114 Republic of Korea; ^4^ Department of Pharmacology College of Dentistry Jeonbuk National University Jeonju 54896 Republic of Korea; ^5^ Department of Mechanical Systems Engineering Sookmyung Women's University Seoul 04310 Republic of Korea; ^6^ School of Chemical and Biological Engineering and Institute of Chemical Process Seoul National University Seoul 08826 Republic of Korea

**Keywords:** biosensors, carbon nanotubes, machine learning, SARS-CoV-2, solution shearing

## Abstract

The recent global spread of COVID‐19 stresses the importance of developing diagnostic testing that is rapid and does not require specialized laboratories. In this regard, nanomaterial thin‐film‐based immunosensors fabricated via solution processing are promising, potentially due to their mass manufacturability, on‐site detection, and high sensitivity that enable direct detection of virus without the need for molecular amplification. However, thus far, thin‐film‐based biosensors have been fabricated without properly analyzing how the thin‐film properties are correlated with the biosensor performance, limiting the understanding of property−performance relationships and the optimization process. Herein, the correlations between various thin‐film properties and the sensitivity of carbon nanotube thin‐film‐based immunosensors are systematically analyzed, through which optimal sensitivity is attained. Sensitivities toward SARS‐CoV‐2 nucleocapsid protein in buffer solution and in the lysed virus are 0.024 [fg/mL]^−1^ and 0.048 [copies/mL]^−1^, respectively, which are sufficient for diagnosing patients in the early stages of COVID‐19. The technique, therefore, can potentially elucidate complex relationships between properties and performance of biosensors, thereby enabling systematic optimization to further advance the applicability of biosensors for accurate and rapid point‐of‐care (POC) diagnosis.

## Graphical abstract

Computational method‐based systematic analysis that can optimize the carbon nanotube (CNT) thin‐film properties and sensitivity of immunosensors is utilized, potentially enabling mass manufacturing of point‐of‐care biosensor for accurate and rapid diagnosis of various diseases. Using CNT films with high alignment and low roughness, nucleocapsid protein in SARS‐CoV‐2 virus is detected with a sensitivity of 0.048 [copies/mL]^−1^.

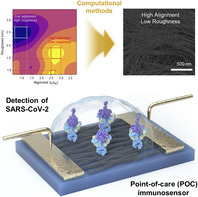

## 1 Introduction

Throughout modern history, the emergence of new viruses and subsequent global outbreak of diseases, such as severe acute respiratory syndrome (SARS), Swine flu, and Middle East respiratory syndrome (MERS), has posed a serious threat to public healthcare.^[^ ^1^ ^]^ Recently, coronavirus disease 2019 (COVID‐19), a disease caused by severe accurate respiratory syndrome‐coronavirus 2 (SARS‐CoV‐2), became a global pandemic. SARS‐CoV‐2, virus containing RNA as the genetic material (i.e., RNA virus), shows more virulent characteristics than DNA virus,^[^ ^2^ ^]^ in which RNA rapidly transcribes and replicates the viral proteins in the infected cells.^[^ ^3^ ^]^ Also, since viral transmission occurs easily through airborne droplets and human‐to‐human contact; rapid diagnosis and quarantine are essential to prevent the spread of the virus.^[^ ^4^ ^]^ However, COVID‐19 patients show asymptomatic or nonspecific symptoms, and the virus is highly contagious even during the incubation period and under asymptomatic conditions, causing indiscriminate viral transmission.^[^ ^5^ ^]^ Therefore, an accurate and rapid diagnosis is of critical importance to control the explosive incidence of COVID‐19.^[^ ^6^ ^]^

Currently, the gold standard for COVID‐19 diagnosis is molecular diagnostics, which detects the viral nucleic acids by amplifying certain genetic sequences of viral genomes with quantitative reverse transcription‐polymerase chain reactions (qRT‐PCRs).^[^ ^7^ ^]^ Medical institutions have steadily used this method due to its high diagnostic accuracy. However, it is time‐consuming and requires laboratory‐based hospitals,^[2b]^ making countries with poor infrastructures struggle to control the spread of virus, eventually leading to increased mortality.^[^ ^8^ ^]^ Thus, it is necessary to develop a low‐cost point‐of‐care (POC) biosensor that detects SARS‐CoV‐2 with rapid detection time and without specialized facilities.

For POC biosensors to be utilizable in clinical applications, the top priority is improving the sensing performance (e.g., sensitivity, limit of detection [LoD]) so that false diagnosis can be reduced.^[^ ^9^ ^]^ Nanomaterial‐based thin films have proven their worth as superior transducers owing to their unique physical and chemical properties.^[^ ^10^ ^]^ For example, their high surface‐to‐volume ratio enhances sensitivity (relative signal change per unit biomarker concentration) by providing a large number of binding sites for bioreceptors.^[^ ^11^ ^]^ Currently, advanced fabrication techniques enable the control of various thin‐film properties (e.g., thickness, surface roughness, alignment, and surface coverage),^[^ ^12^ ^]^ through which the biosensor's performance can be optimized. In this regard, we have recently reported carbon nanotubes (CNTs)‐based biosensors that can diagnose Alzheimer's disease via multiplexed detection of biomarkers in human plasma.^[^ ^13^ ^]^ Specifically, densely aligned CNT films were produced by the Langmuir−Blodgett (LB) method, which allowed detection of targeted biomarkers down to femtomolar concentrations. Also, research on improving biosensing performance by optimizing thin‐film density and the length and width of hydrothermally grown ZnO nanowires was reported,^[^ ^14^ ^]^ where tuning such properties improved sensitivity for the detection of SARS‐CoV‐2 virus.

However, so far, the development of nanomaterial thin‐film‐based biosensors has been conducted without fully understanding how and to what degree the aforementioned thin‐film properties affect the sensing performance of the biosensor, which has thus far likely limited the optimization of sensing performance. Therefore, it is of critical importance to develop a systematic approach to understand how the sensing performance is correlated with each of the thin‐film properties and what combination of thin‐film properties presents the highest sensing performance.

## 2 Results and Discussion

**Figure 1:**
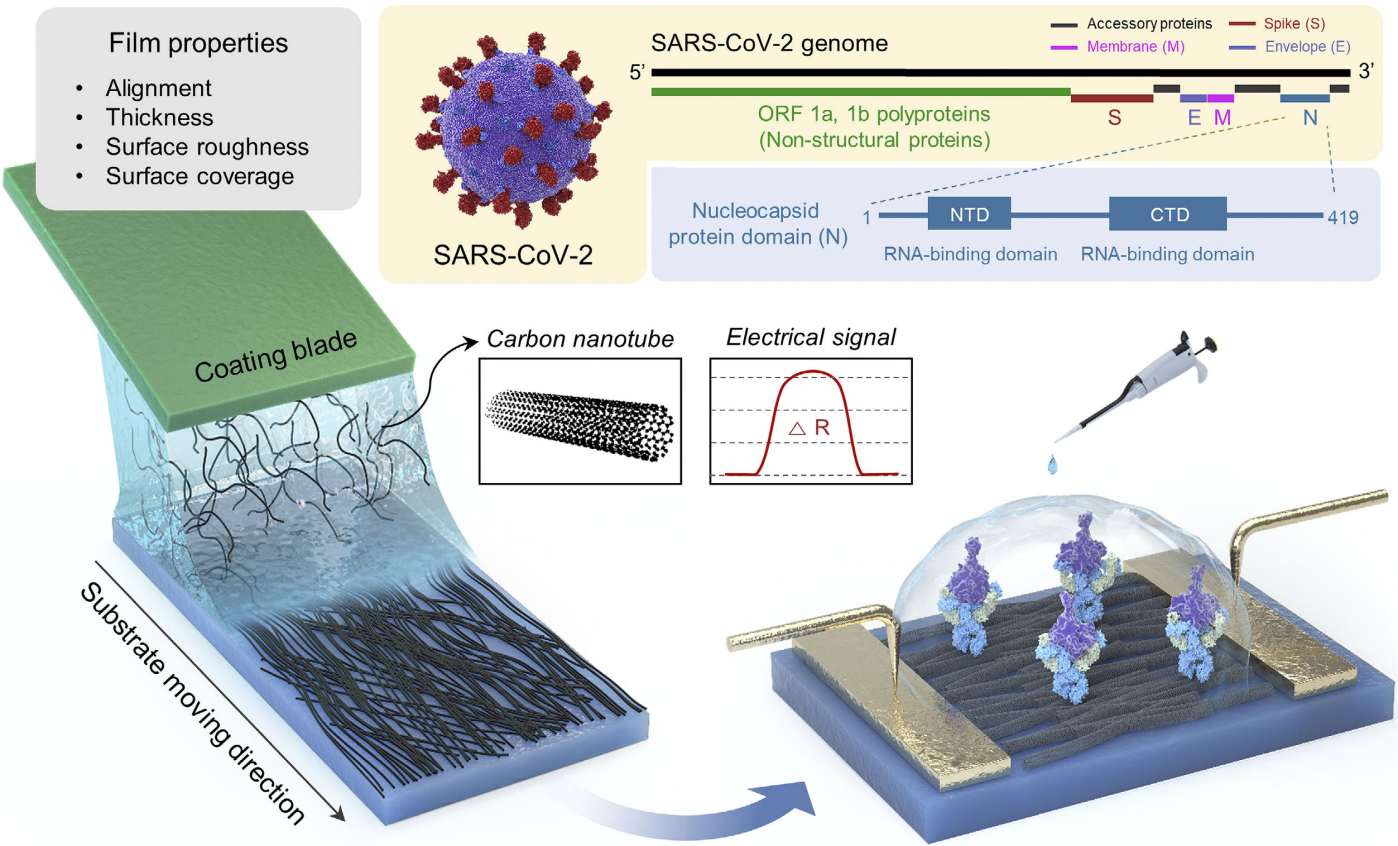
Overall schematic illustration of fabricating CNT thin‐film‐based resistive immunosensors. Thin‐film properties affecting biosensing performance can be tuned using experimental parameters, through which sensitivity can be optimized. By optimizing the sensitivity, NP in SARS‐CoV‐2 virus can be detected in a rapid and reliable manner.

In this work, we have utilized solution shearing to regulate the thin‐film properties of CNTs (a commonly utilized nanomaterial for biosensing),^[^ ^15^ ^]^ which potentially enables mass manufacturing of the POC biosensor at a low cost (**Figure** **1**).^[^ ^16^ ^]^ In solution shearing, solution with uniformly dispersed CNTs forms a meniscus between a heated substrate and a coating blade.^[^ ^17^ ^]^ As the blade moves, continuous deposition of CNTs occurs near the edge of the meniscus (owing to the accelerated solvent evaporation),^[^ ^18^ ^]^ forming a thin film. Using the CNT thin film as the sensing surface, resistive biosensors were fabricated to detect immunoglobulin G (IgG) and SARS‐CoV‐2 antigen protein, where thin‐film properties, such as alignment, thickness, surface roughness, and surface coverage, were tuned. Herein, machine learning models were utilized to analyze the relative importance and the correlation of these thin‐film properties toward the sensitivity of the biosensor, through which sensitivity was optimized. By this methodology, CNT‐based biosensors were able to detect IgG and SARS‐CoV‐2 nucleocapsid proteins (NPs) in both buffer solution and lysed virus with a sensitivity of 0.093 fM^−1^, 0.024 [fg/mL]^−1^, and 0.048 [copies/mL]^−1^, respectively. Our machine learning‐assisted systematic analysis not only enables optimization of biosensors but also allows evaluation of the relative importance and correlation of various thin‐film properties, potentially expanding the applicability of biosensors toward POC testing for accurate and rapid diagnosis of various diseases.

**Figure 2:**
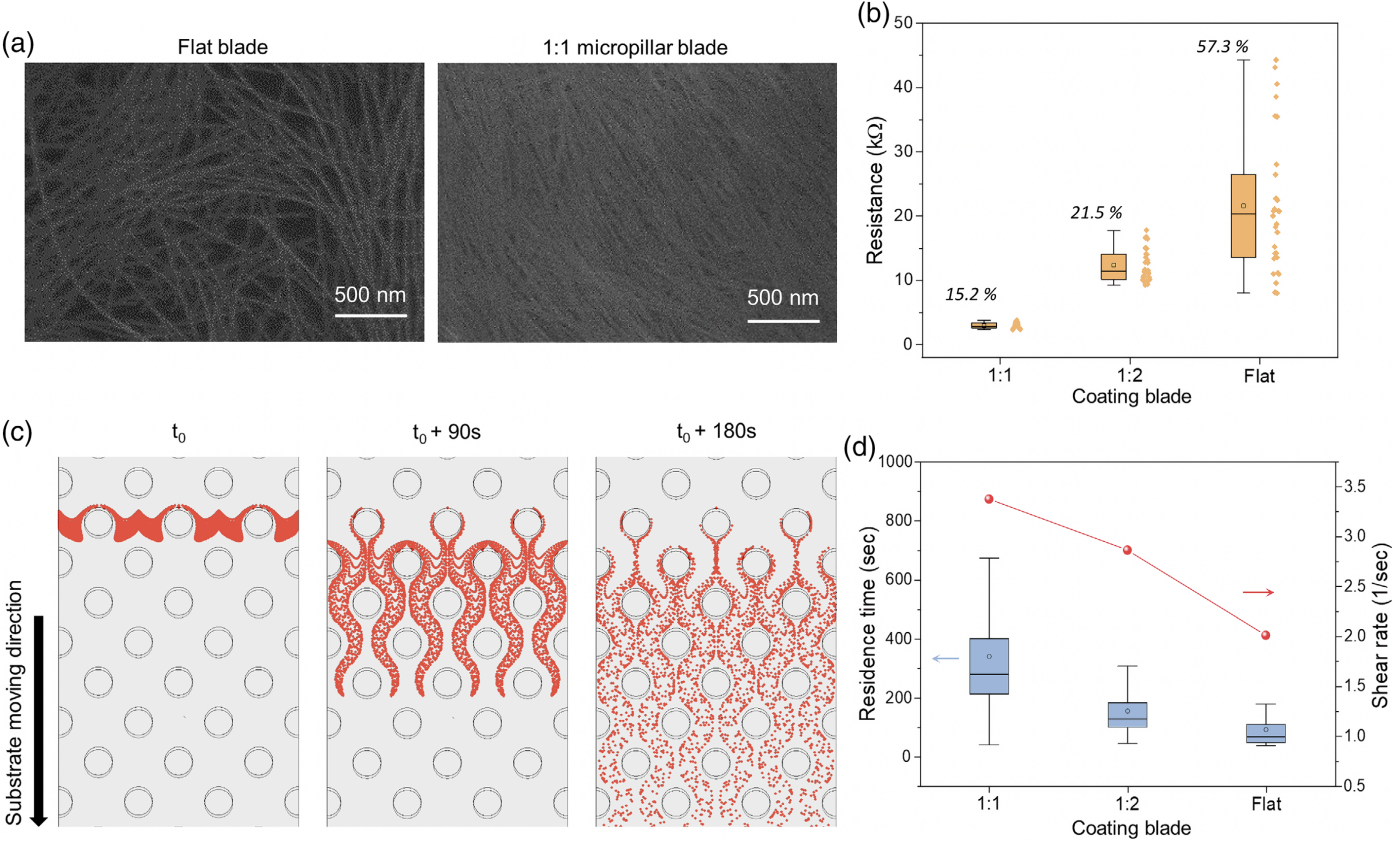
Comparison of morphology and electrical properties of the CNT thin films fabricated by flat and microstructured (1:1, 1:2 spacing) blades. a) SEM images of CNT films fabricated by flat (left) and 1:1 spacing (right) microstructured blades. The microstructures alter the fluid behavior, resulting in the highly packed CNT films. b) Comparison of CNT thin‐film resistance. CV values (italicized) were four times lower in the 1:1 spacing microstructured blade than in flat blade. c) Numerical simulation visualizing trajectories of particles around the micropillars (1:1 spacing) using forward particle tracking methods. d) Calculation of residence time and shear rate for aforementioned three coating blades, showing the broadest distribution of residence time and the highest shear rate in 1:1 spacing microstructured blade.

Fluid flow behavior during solution shearing plays a pivotal role in determining the thin‐film properties as it affects solute transport and liquid‐to‐solid transition.^[^ ^19^ ^]^ Previously, solution shearing blade was microstructured to manipulate flow behavior, through which organic thin‐film crystallization was controlled for tuning the performance of thin‐film transistors^[18b]^ and solar cells.^[^ ^20^ ^]^ In this work, we utilized microstructured blade as a means to generate highly uniform and low‐resistance CNT films, which has not been demonstrated before. Here, three different blades were used to generate CNT thin films, all under the same shearing condition (see Experimental Section for details). First, blade without microstructuring was used, which we will call flat blade here onward. Next, two different microstructured blades were used. Both blades had circle‐shaped pillars with height and diameter of 20 μm, arranged as body‐centered cubic (BCC), but with different edge‐to‐edge spacing of 20 μm (1:1 ratio of diameter to spacing) and 40 μm (1:2 ratio of diameter to spacing). For these three blades, we compared the difference in fluid characteristics using numerical simulation and analyzed the CNT film properties (see Figure S1 and Videos S1–3 in the Supporting Information for further details). As shown in the scanning electron microscopy (SEM) image in **Figure** **2**a, the CNT thin film generated with 1:1 blade had a much more densely and uniformly covered surface compared with that of the flat blade. The surface roughness for the 1:1 blade film was also relatively low (*R*_a_: 1.54 nm) compared with that of the flat blade (*R*_a_: 3.01 nm). The resistances of the three types of films were measured, each at 40 different locations using patterned electrodes, as shown in Figure 2b. The film generated with 1:1 blade had the highest uniformity (coefficient of variation [CV] of 15.2%) and the lowest average resistance of 2.95 kΩ. On the contrary, the flat blade resulted in the lowest uniformity and the highest average resistance. We therefore hypothesized that microstructuring prevents aggregation/bundling of CNTs and spatially spreads out the CNTs uniformly in the solution. Figure 2c shows a numerical simulation showing the particle trajectories, in which particles are initially assembled perpendicular to the coating direction and pass through the 1:1 blade. BCC‐arranged microstructures disrupt the laminar flow and accelerate the fluid flow in the perpendicular direction, affecting the solute transport to the liquid−solid boundary. Figure 2d shows the residence time of the simulated particles (i.e., time taken for a given particle to pass through a designated region) and calculated shear rate at the tip of the blade for the three types of blades (see Experimental Section for further details on simulation). The 1:1 blade had the highest of such values, whereas, the flat blade had the lowest of such values, which was the same trend observed for uniformity and average resistance. We postulate that higher shear rate breaks up, prevents CNT aggregation, and enhances CNT alignment.^[^ ^21^ ^]^ Furthermore, the wide distribution of residence time likely enhances the spatial distribution of CNTs in the solution, which increases film uniformity. Further analysis is required to confirm these hypotheses and is gcurrently the subject of our future work. For the rest of the experiments presented in this report, 1:1 blade was used.

**Figure 3:**
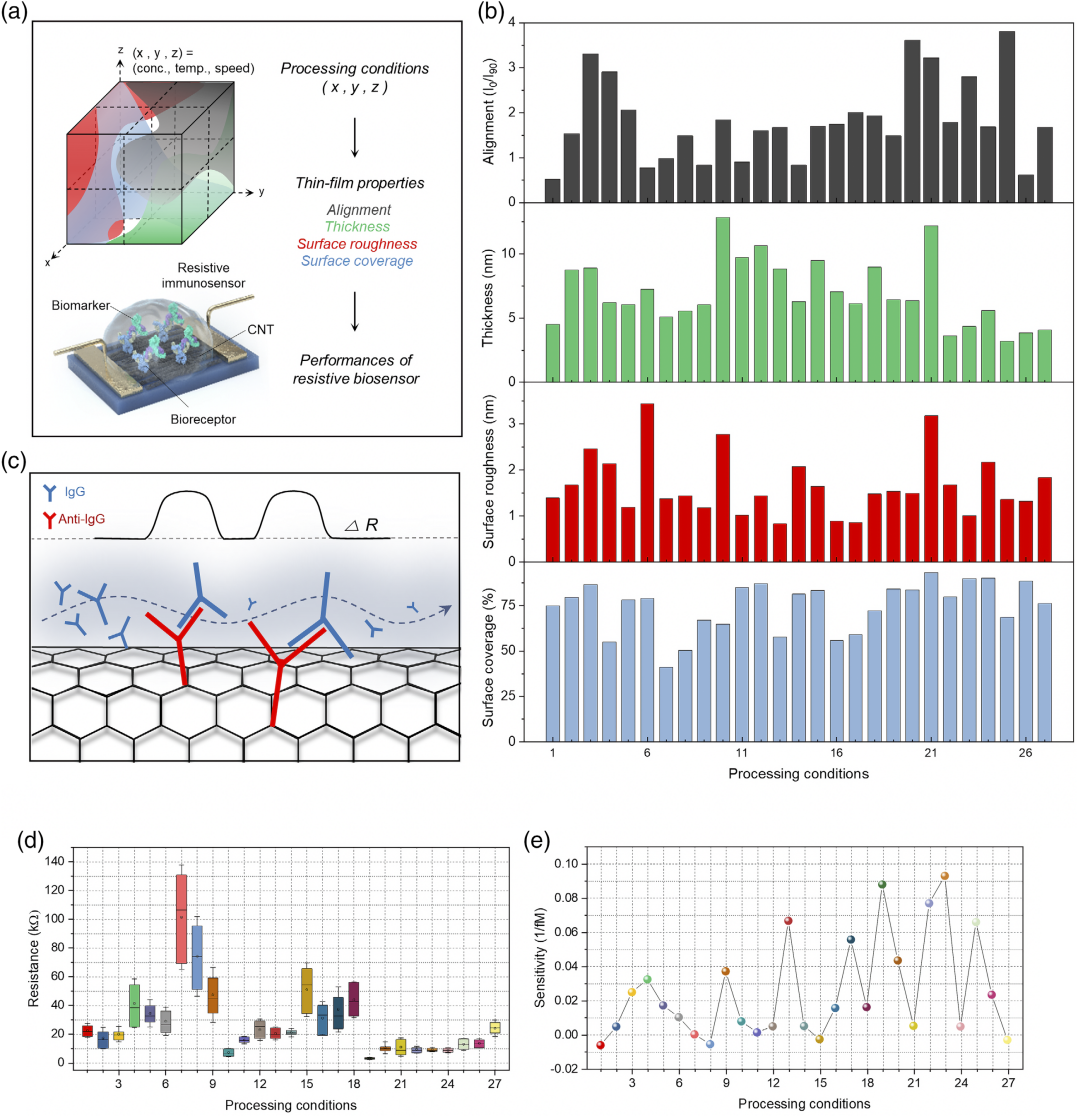
Thin‐film properties and sensitivities of the CNT‐based resistive immunosensors depending on the processing conditions. a) Schematic depicting the relationships between the processing conditions, thin‐film properties, and biosensor performances. Processing parameters are represented as *x, y, z*. b) Changes in four thin‐film properties (alignment, thickness, surface roughness, surface coverage) depending on the processing conditions. c) Schematic illustration of the resistive biosensing mechanism due to antigen−antibody binding. d) Comparison of CNT thin‐film average initial resistance. Each data is based on 40 devices. The whiskers represent mean ± standard deviation. e) Comparison of CNT sensor sensitivity toward IgG. Each data point represents the average sensitivity from two different CNT sensors, showing both positive and negative correlation with regard to processing conditions.

To tune the properties of the thin film, three processing parameters were varied: CNT solution concentration, coating speed, and substrate temperature. Thin film was generated at various values of these processing parameters, resulting in a total of 27 processing conditions. Here, “processing condition” refers to a set of specific values of processing parameters under which the thin film was formed (see Table S1 from the Supporting Information). Graphically, the three processing parameters represent three different axes and points in this cuboidal parameter space are the processing conditions (**Figure** **3**a). At each condition, thin‐film properties (thickness, surface roughness, surface coverage, and degree of alignment) were measured. How these thin‐film properties were measured are detailed in Experimental Section and Figure S2,S3 from the Supporting Information. As shown in Figure 3b, each of the thin‐film properties exhibited different values and trends as a function of processing conditions.

Next, resistive immunosensors were fabricated using the thin films generated under the aforementioned processing conditions. Here, immunoglobulin G (IgG) was set as the target biomarker; hence, the corresponding antibody (anti‐IgG) was anchored on the surface of the CNTs (see Experimental Section and Figure S4 in the Supporting Information for details on the fabrication process). As a complex is formed by coupling between IgG and anti‐IgG, resistance of the film increases (Figure 3c) due to the charge scattering effect.^[15b]^ As shown in Figures 3d,e, the thin films generated under different conditions exhibited significant differences in the average initial resistance *R*_o_ (i.e., resistance prior to the binding of biomarkers in buffer solution) and sensitivity (i.e., slope of the relative change in resistance [Δ*R*/*R*_o_] versus biomarker concentration, expressed in 1/*fM*). The reason for the differences in the initial resistances and sensitivities at each condition is due to the differences in the thin‐film properties. For instance, looking closely at the data in Figure 3d,e, conditions 19 and 23 had the two highest sensitivities, and at these conditions, relatively low initial resistance and CV were attained. Such a correlation can be attributed to reduced bundling and better alignment of CNTs; these affects will be discussed further. However, due to the presence of multiple thin‐film properties, it is not obvious from observing the data in Figure 3b–e the relative importance of each thin‐film property and what combination of thin‐film properties results in higher performance. Therefore, for device optimization and for better understanding of property−performance correlation, machine learning‐assisted computation needs to be utilized.

**Figure 4:**
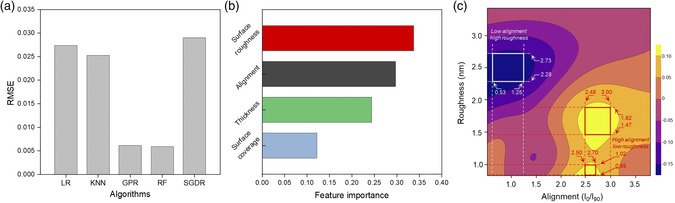
Correlation between the thin‐film property and sensitivity by machine learning‐based computation. a) Comparison of performance of different machine learning models (e.g., LR, KNN, GPR, RF, and SGDR) using the cross‐validated RMSE. The inputs are normalized. b) Feature importance of various CNT thin‐film properties (surface roughness, alignment, thickness, and surface coverage) using the random forest model. c) Prediction map of sensitivity generated using GPR model, where the range of roughness and alignment yielding high sensitivity (yellow region) and low sensitivity (purple region) was attained. *x*‐axis represents degree of alignment and *y*‐axis represents surface roughness, at the thickness of 4.34 nm.

Using the aforementioned data, machine learning‐based computation was conducted to extract the correlation between thin‐film properties (input variables) and sensitivity (target variable).^[^ ^22^ ^]^ Details of the computation process are shown in Note S1, Supporting Information. We tested five machine learning models to predict the sensitivity using the scikit‐learn library in Python: linear regression (LR), *K*‐nearest neighbors (KNN), Gaussian process regression (GPR), random forest regression (RF), and stochastic gradient descent regression (SGDR). The *k*‐fold cross‐validation (*k* = 5) was used to verify the predictive accuracy for each of the models. **Figure** **4**a shows the cross‐validated root mean square error (RMSE) values of the models, indicating that GPR and RF show lower RMSE compared with that of other algorithms without the evidence of overfitting (see Table S2 in Supporting Information).^[^ ^23^ ^]^ GPR is a nonparametric kernel‐based model that provides a probabilistic estimate of the approximation,^[^ ^24^ ^]^ and RF is an ensemble model with several decision trees,^[^ ^25^ ^]^ where estimators are predicted from a subset of data. These models allow the management of complex correlations between input and target variables. In this study, GPR model was utilized for predicting the sensitivity while RF model was used to investigate feature contribution, as shown in Figure 4b.

Figure 4b shows the result of feature contribution analysis to test the degree of importance of each thin‐film property to the sensitivity (thin‐film properties will also be referred to as “features” from this point onward). The surface roughness, alignment, and thickness contribute to about 0.34, 0.30, and 0.24, respectively, whereas, the surface coverage only accounts for a contribution of 0.12. Therefore, surface coverage was omitted as a feature for our study. The low contribution of surface coverage is attributed to the fact that solution‐sheared CNT films generated under our processing conditions show low variability in surface coverage than that of the other film properties (Figure S5 in the Supporting Information). Figure S6 in the Supporting Information shows a 3D map that predicts the sensitivity in a cuboidal feature space, where the axes represent the other three thin‐film properties (e.g., surface roughness [*x*‐axis], thickness [*y*‐axis], and alignment [*z*‐axis]). Figure 4c shows a 2D slice of the 3D map at a specific value of thickness (which was the least influential feature out of the three features) that contains the highest predicted sensitivity, where *x*‐ and *y*‐axis represent alignment and surface roughness, respectively (i.e., this slice does not contain the experimentally attained sensitivity data points). It is evident that higher sensitivity (yellow region) is predicted at low surface roughness and high CNT alignment; vice versa is true for attaining lower sensitivity (purple region). Within these regions of extrema, rectangular areas were defined, which consist of ranges of alignment and surface roughness values.

**Figure 5:**
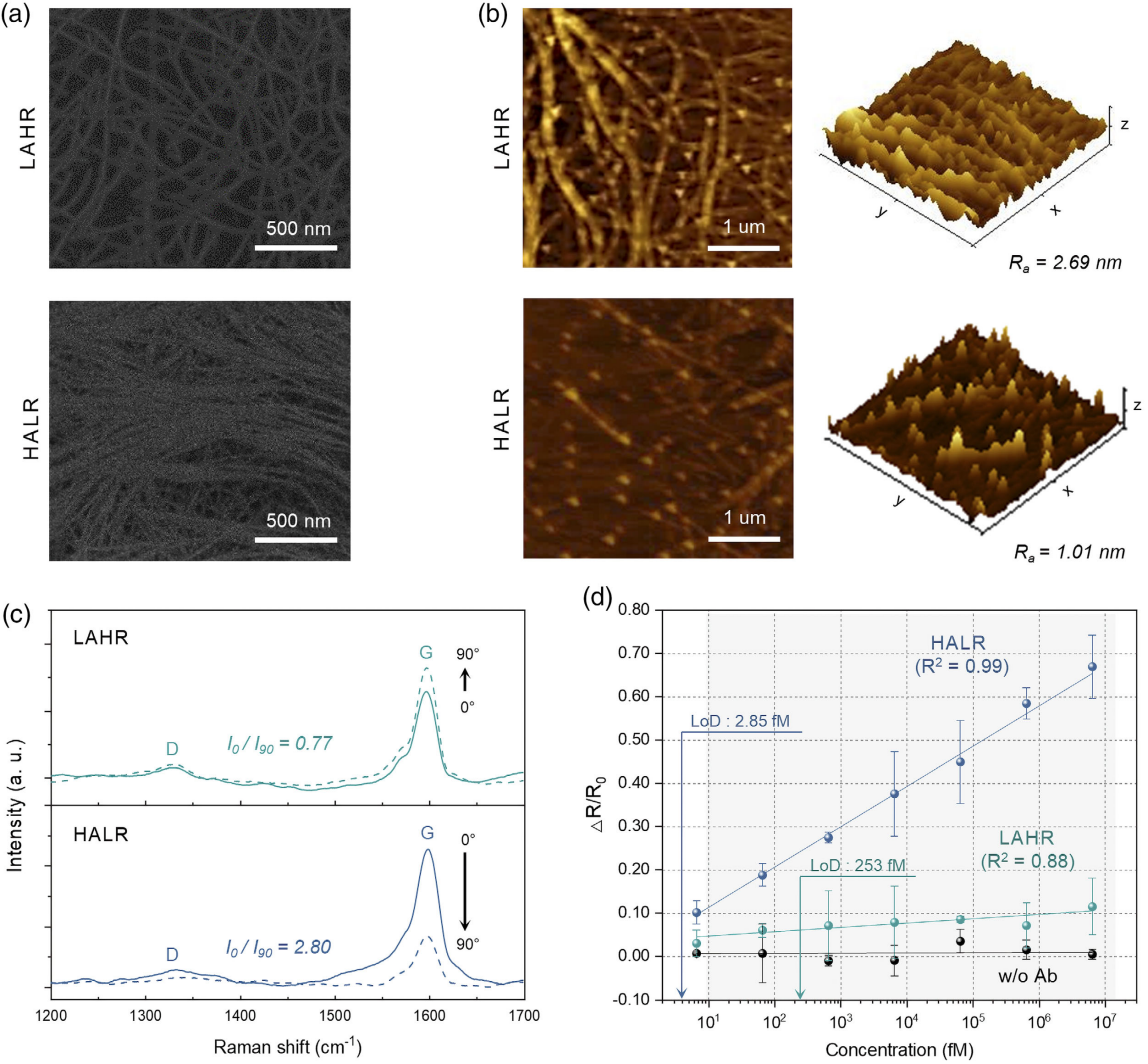
Characterization and sensitivities of two types of CNT‐based immunosensor. a,b) SEM images (a) and AFM images (b) of LAHR‐ and HALR‐based CNT thin‐films. The two CNT films show different values of surface roughness (italicized). c) Polarized Raman spectra of CNT films. The data show Raman intensity at 1595 cm^−1^ depending on the angle difference between 633 nm incident laser and the coating direction of CNT films. The alignment of CNT films (italicized) was calculated by the ratio of the G‐band intensity at rotating angles of 0° (laser incident parallel to coating direction) and 90° (laser incident perpendicular to coating direction). d) Sensing performance of different types of CNT thin films. For each data point, a different set of four sensors was used. The negative control was conducted without anti‐IgG. All the values represent the mean ± standard deviation.

The decrease in sensitivity with increasing surface roughness can be explained by the decrease in surface area available for antibody binding. High surface roughness is attributed to increased CNT bundling, as shown in **Figure** **5**b. As the CNTs within the inner layer of the bundles are not accessible for antibody binding, the number of bound antibodies per total CNT surface area is reduced, which consequently reduces the sensitivity. Also, bundling has been shown to increase the resistance of the film as current mainly flows along the outermost CNTs of bundles rather than the inner CNTs.^[^ ^26^ ^]^ Since complex formation is detected by the relative change in the resistance of the film,^[^ 13, 15 ^]^ increased initial resistance of the film lowers the sensitivity. The increase in sensitivity with increasing alignment can be explained by the decrease in tube‐to‐tube junctions. Tube‐to‐tube junctions have been determined to be much more resistive compared with that of resistance along CNTs.^[26a]^ Therefore, for misaligned films, as the resistance of the film is dominated by the resistance of the tube‐to‐tube junctions, we postulate that tube‐to‐tube junctions effectively “hide” the change in resistance due to complex formation. Verifying these hypotheses requires further investigation and is the subject of our future work.

To demonstrate the validity of the predicted correlation between thin‐film properties and sensitivity, two types of thin films were made, one with properties satisfying the high‐sensitivity yellow region (High Alignment Low Roughness [HALR]) and another satisfying the low‐sensitivity purple region (Low Alignment High Roughness [LAHR]). To find the processing conditions that would result in these thin‐film properties, GPR model‐based machine learning was utilized to find the correlation between the processing conditions (input variables) and each of the three thin‐film properties (target variables: roughness, alignment, and thickness). In other words, three cuboidal parameter space models were generated where the axes were the three processing parameters. Thereafter, processing conditions that yielded a proper range of values for each of the thin‐film properties were determined. Next, processing conditions that simultaneously satisfied the range of values for all three thin‐film properties were deduced (see Figure S7 in the Supporting Information for details).

Figure 5a shows SEM images of the two types of CNT thin films, which we define as “LAHR” and “HALR.” Figure 5b shows the atomic force microscopy (AFM) topographic images, demonstrating that LAHR had a higher mean roughness *R*_a_ (2.69 nm) compared with HALR (1.01 nm), and these values were indeed within the range of roughness values shown in Figure 4c. Next, the degree of alignment was measured by taking the ratio of G‐band intensities in Raman spectra at rotation angles 0° and 90° (Figure 5c). For LAHR, the degree of alignment was 0.77, while that of the HALR was 2.80. These values again were within the range of alignment values shown in Figure 4c. Furthermore, using these two films, resistive immunosensors were fabricated, and their sensitivities toward IgG were calculated by measuring the relative change in resistance at various IgG concentrations in buffer solution (from 10^1^ to 10^7^ fM) and taking slope of the regression line. Low sensitivity was attained for the LAHR‐based sensor (0.010 fM^−1^), whereas, HALR‐based sensor had relatively high sensitivity (0.093 fM^−1^), which was 9.3 times higher. Moreover, for the HALR‐based sensor, the coefficient of determination (*R* ^2^) of the regression line was relatively high (0.99), and the CV value of the initial resistance was relatively low compared with that of the LAHR‐based sensor (Figure S8 in the Supporting Information). The LoD for the HALR‐based sensor estimated at a confidence level of 3.3 was 2.85 fM, which was 88.8 times lower than that of the LAHR‐based sensor (Figure S9a in the Supporting Information for details). These results show that machine learning can predict the resulting thin‐film properties from a given set of processing parameters, through which optimal biosensor performance can be obtained in an efficient manner.

To verify whether the trend in the sensor performance obtained earlier translates to other biomarkers and to confirm the viability of our sensor toward diagnosis of COVID‐19, the two types of films were utilized to detect SARS‐CoV‐2 NPs in PBS solution. To select an antibody for detecting the NP antigen, first, we compared the sensitivity and selectivity of four single‐chain variable fragment‐crystallizable (scFv‐Fc) antibodies and we developed in a previous report by indirect ELISA assay (Figure S10 in Supporting Information).^[^ ^27^ ^]^ 12H1 and 12H8 antibodies showed high sensitivity, and 12H1 antibody showed the best selectivity (Figure S11 in the Supporting Information). Thus, 12H1 scFv‐Fc antibody was used in CNT sensors.

**Figure 6:**
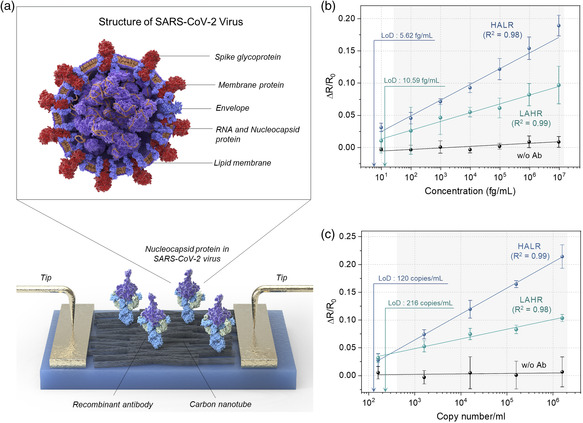
Sensitivities of the CNT‐based immunosensors toward SARS‐CoV‐2 NPs. a) The structure of SARS‐CoV‐2 virus and schematic illustration of CNT film‐based resistive immunosensor detecting SARS‐CoV‐2 NPs. b,c) Relative change in the resistances of CNT sensors with LAHR‐based thin film and HALR‐based thin film upon exposure to SARS‐CoV‐2 NPs in buffer solution (b) and in the lysed SARS‐CoV‐2 virus (c). Sensors without scFv fusion protein in SARS‐CoV‐2 NP conjugation were used as a negative control. For each data point, a different set of four sensors was used. All the values represent the mean ± standard deviation.

**Figure** **6**a shows the schematic depiction of the SARS‐CoV‐2 virus with labeling of its components. As shown in Figure 6b, for both types of CNT sensors, we observed a strong linear relationship between the relative resistance change and NP concentration in the range from 10 fg/ml to 10 ng/ml, where the coefficients of determination (*R* ^2^) of the regression line were over 0.98. Similar to the sensitivity trend shown in Figure 5d for IgG, the HALR‐based sensor exhibited relatively high sensitivity toward SARS‐CoV‐2 NP; the sensitivity was 1.71 times higher than that of the LAHR‐based sensor. The estimated LoD values of the HALR‐ and the LAHR‐based sensor at a confidence level of 3.3 were 5.62 and 10.59 fg/ml, respectively.

We further confirmed the potential of our sensors for rapid diagnosis by directly detecting NPs in the SARS‐CoV‐2 virus, as shown in Figure 6c. SARS‐CoV‐2 viruses were cultured in Dulbecco's minimal essential medium (DMEM). Then, the cultured viruses were lysed, and the NPs in viruses were detected. Therefore, Figure 6b,c show sensitivities toward NPs, while in the case of Figure 6b, pure NP solution without viral impurities was used. Again, to obtain the sensitivity and LoD of the two types of sensors, relative resistance change as a function of cultured SARS‐CoV‐2 virus concentration from 1.62 × 10^2^ to 1.62 × 10^6^ copies/ml was measured. The results show that the HALR‐based sensor also exhibited relatively high sensitivity toward the NPs in the SARS‐CoV‐2 virus; the sensitivity was 2.76 times higher than that of the LAHR‐based sensor. Moreover, the LoDs of the HALR and LAHR‐based sensor were estimated to be 120 and 216 copies/ml, respectively, at a confidence level of 3.3 (Figure S9b in the Supporting Information for details). Based on the detection of NP, the attained LoD of the HALR‐based sensor strongly correlates with the LoD shown in Figure 6b (5.62 fg/ml). Details of the calculation are shown in Note S2, Supporting Information. These results show that the HALR‐based sensor can reliably detect SARS‐CoV‐2 virus at a level of about 100 copies without being disturbed by interfering agents in virus culture medium (Figure S12 in the Supporting Information). It is known that SARS‐CoV‐2 virus is found within the first week after symptom onset,^[^ ^28^ ^]^ to a viral load of around >10^6^ copies/ml,^[^ 28, 29 ^]^ which was set as a detection cutoff for antigen‐detecting rapid diagnostic tests by WHO.^[^ ^30^ ^]^ Furthermore, recent literatures mentioned that the viral loads of SARS‐CoV‐2 in clinical samples by RT‐PCR ranged from 6.40 × 10^2^ copies/ml to 1.35 × 10^11^ copies/ml, with a median of 1.69 × 10^5^ copies/ml for nasal swab samples, 7.99 × 10^4^ for throat samples, and 7.52 × 10^5^ for sputum samples, taken 3 days after symptom onset.^[^ ^31^ ^]^ Therefore, the LoD of our sensor with about 100 copies/ml is lower than the viral load in clinical samples. This sensor also showed selectivity by accurately discriminating the NPs of SARS‐CoV‐2, compared with other types of biomarkers, such as NPs of MERS‐CoV and Influenza A (Figure S13 in the Supporting information). This indicates that our HALR‐based sensor fulfills the sensitivity requirement and could potentially be used to diagnose patients in the early stages of COVID‐19.

## 3 Conclusions

In this work, CNT thin‐film‐based biosensor was optimized via investigating the correlation between thin‐film properties and sensitivity using machine learning‐based computation. Our approach enables systematic optimization of sensor performance rather than relying on human intuition, which has greater potential for attaining higher sensor performance. At a given film thickness, smaller surface roughness and better CNT alignment yielded higher sensitivity, which can be attributed to increased surface area for bioreceptor binding and lower density of tube‐to‐tube junctions. Our optimized sensors showed sufficiently high sensitivity for detecting NPs in the SARS‐CoV‐2 virus in patients in the early stages of COVID‐19. We project that our computational approach can be generally applied to other sensing systems to elucidate the complex relationships between properties and performance, through which optimal device performance can be found in an efficient manner, thus expanding the applicability of various biosensors for accurate and rapid diagnosis.

## Conflict of Interest

The authors declare no conflict of interest.

## Supporting information

Supplementary Material

## Data Availability

Research data are not shared.
