# Supplementary material for: Computational Method‐Based Optimization of Carbon Nanotube Thin‐Film Immunosensor for Rapid Detection of SARS‐CoV‐2 Virus
Source: Small Sci. 2021 Nov 16;2(2):2100111. doi: 10.1002/smsc.202100111 (PMC8646396; doi:10.1002/smsc.202100111)
Supplement: Supplementary file 1 — Supplementary Material [file SMSC-2-2100111-s001.zip › Supporting Information.pdf]

Supporting Information

Computational method-based optimization of carbon nanotube thin-film immunosensor for rapid detection of SARS-CoV-2 virus

Su Yeong Kim, Jeong-Chan Lee, Giwan Seo, Jun Hee Woo, Minho Lee, Jaewook Nam, Joo Yong Sim, Hyung-Ryong Kim, Edmond Changkyun Park,* Steve Park**

Experimental section

Materials

Allylhydropolycarbosilane (AHPCS, SMP10), Photo-initiator (Igacure 369), and dicumyl peroxide thermal initiator used to fabricate the microstructured blade were purchased from Starfire Systems Inc. (Malta, NY, USA), Ciba Specialty Chemicals Inc, Sigma-Aldrich, respectively. Single-walled carbon nanotube (SWCNT) was obtained from OCSiAl, USA. Other chemical reactants were purchased from Sigma-Aldrich and used without any further purification.

Fabrication of microstructured blade and solution shearing

To fabricate the PDMS mold, a Si wafer was microstructured using the negative photoresist (SU-8, MicroChem, USA). The 10:1 mixture of PDMS:curing agent was cured onto the patterned wafer for 2 hours at 80 °C. The patterned PDMS mold was treated with trichloro-(1H,1H,2H,2H-perfluorooctyl)silane by chemical vapor deposition (CVD) at 70 °C for 30 min. Inorganic polymer resin, AHPCS, was dropped onto a Si wafer substrate, and partially cured with UV exposure (50 mW cm^{-2} for 3 min) and thermally cured for 4 hours at 120 °C.[1] To make the surface hydrophobic, the microstructures were hydrolyzed in 0.5 M NaOH solution for 10 min at 70 °C and treated with trichloro-(1H,1H,2H,2H-perfluorooctyl)silane by CVD for 30 min at 70 °C. Using the microstructured blades (1:1, 1:2 spacing) and the flat

blade, CNT solution was sheared under the same coating condition (concentration: 1 mg/ml, coating speed: 0.1 mm/s, temperature: 55 °C).

Numerical simulation for fluid behavior

The numerical simulations used to analyze the microstructured blade system were analyzed by the stabilized space-time Galerkin/least-squares methods using in-house software (eXecutable Navier-Stokes, XNS).[2] The solution with uniformly dispersed CNTs was considered as an incompressible, Newtonian fluid with constant density, viscosity, and kinematic viscosity. All computations were performed on a DELL PowerEdge R 730 and R640 cluster with 480 CPUs and 2560GB of memory. Forward particle tracking method was used to visualize the trajectories of fluid particles in the flow field.

Machine learning model building

The machine learning models were built by the Python language in the PyCharm IDpE. We used various Python side packages (e.g., numpy, pandas, matplotlib, scikit-learn, shap, and plotly) to preprocess, visualize, and train prediction models. Linear-Regression (LR), Stochastic Gradient Descent Regression(SGDR), Random Forest (RF), Gaussian Process Regression (GPR), and K-Nearest Neighbors (KNN) were used with default hyper-parameters of scikit-learn to predict the sensitivity. In the training, the data were randomly split (80 %:20 % training:test dataset). To prevent the overfitting within a limited data sample, we have performed the 5-fold cross-validation for all algorithms (Table S2). The feature importance for thin-film properties was conducted using Random Forest algorithm.

Solution shearing

The 1:1.2 ratio of CNT: poly (3-dodecylthiophene-2,5-diyl) (P3DDT) solution was prepared at a concentration of 0.5, 0.75, and 1 mg/ml based on CNT concentration. The tilt of the coating blade was 8 °. Coating speed, determined by substrate moving speed relative to the stationary blade, was 0.1, 0.3, and 0.5 mm/s. Substrate temperature was 55, 70, and 85 °C. The processing conditions were designed using the coating speed, the substrate temperature, and the concentration of CNT solution (Table S1).

Thin-film characterization

Thin-film properties (thickness, surface roughness, surface coverage, and alignment) of solution-sheared CNT films were characterized to investigate their effects on sensing performance. Film thickness was calculated by measuring the difference in height value between the CNTs-coated region and bare region through Alpha-Step (Alpha-Step D500, KLA Co., USA). Details of the calculation are in Figure S2. Surface roughness was measured using tapping mode AFM (AFM WORKSHOP, PS-2010), and alignment was calculated by the ratio of the G-band intensity at rotating angle of 0° and 90° using LabRAM HR Evolution, HORIBA. Also, SEM images were used to calculate surface coverage, which was quantified via the image J program and verified by the difference in fluorescence intensity (Figure S3).

Fabrication of carbon nanotube sensor

The as-prepared CNT film was annealed at 500 °C for 1 hour to remove P3DDT moieties. We patterned the CNT sensor array by depositing metallic source and drain contacts (Au/Cr = 50/5 nm) on top of the CNT film with an e-beam evaporator (SNTEK Co. Ltd, Korea). The sensor array was patterned in two rows, and the width and the length of each CNT channel

were 1.0 mm and 100 μm, respectively. Each array contained 40 devices to measure the average resistance, and 4 devices per biomarker concentration were used to get the sensitivity.

Functionalization and immobilization of carbon nanotube film

To carboxylate the surface of the CNT film, we treated the as-fabricated CNT sensor array with O_2 plasma. After exposing O_2 plasma for 25 sec, we mounted the PDMS well on the as-treated CNT sensor array to diffuse the analyte only on top of the CNT channel and avoid binding of analytes on the unwanted area. Each CNT sensor was incubated in 0.1 M 2-(*N*-Morpholino)ethanesulfonic acid (MES) buffer (pH 5.0) solution containing 200 mM 1-Ethyl-3-(3-dimethylaminopropyl)carbodiimide (EDC) and 500 mM *N*-hydroxysuccinimide (NHS) for 30 min at room temperature. Then, we washed each CNT sensor with 10 mM phosphate buffer saline (PBS, pH 7.4) solution to remove the residual chemicals. To immobilize the antibody, the 300 μg/mL of anti-IgG and scFv fusion protein in SARS-CoV-2 NP were dropped on the EDC/NHS-treated CNT channels, and incubated overnight at 4 °C. After that, we washed the CNT sensor array with 10 mM PBS solution composing Tween 20 of 0.5 wt.% to remove the unbound antibodies from the channels. We subsequently added the 10 mM PBS solution of 3 wt.% bovine serum albumin (BSA) and 0.5 wt.% Tween 20 to each CNT channel for 1.5 hours at 4 °C, to prevent non-specific binding. Then, we washed each CNT channel with 10 mM PBS solution containing 0.5 wt.% Tween 20 to remove the unbound materials.

Procedure of preparing biomarkers (IgG, SARS-CoV-2 NP antigen), antibodies, and cultured viruses

We prepared the solutions of IgG ranging from femtomolar to nanomolar concentration by serially dissolving the biomarkers in 10 mM PBS solution. Each IgG solution was uniformly

dispersed through centrifugation. SARS-CoV-2 NP antigen and its antibodies were prepared as described in the previous report.[3] In brief, for the antigen protein, the full-length SARS-CoV-2 NP gene was synthesized and cloned into a pET28a plasmid. Then the construct was transformed into *E. coli* BL21, and expressed recombinant SARS-CoV-2 NP was purified from the bacterial cell lysate by affinity chromatography and a sized-exclusion column. For the antibody, four SARS-CoV-2 NP-specific single-chain variable fragment (scFv) clones were isolated from a chicken naïve phage library by biopanning and inserted into single-chain variable fragment-crystallizable (scFv-Fc) plasmid. Then, the SARS-CoV-2 NP scFv-Fc antibodies were expressed in 293F cells and purified by protein A affinity chromatography. To culture SARS-CoV-2 virus, virus culture was conducted in a biosafety level 3 laboratory. Vero E6 cells were infected with SARS-CoV-2 (BetaCoV/Korea/KCDC03/2020, provided by Korea CDC), After 48 hours, the culture medium containing mature infectious virions was collected, and the viral titer was measured by RT-PCR. The live virus was inactivated by heating to 100 °C for 15 min and then stored at -80 °C until required. The samples were serially prepared from 1.62×10^2 copies/mL to 1.62×10^6 copies/mL.

Indirect enzyme-linked immunosorbent assay (ELISA)

A 96-well plate was coated with antigens from SARS-CoV-2, SARS-CoV, MERS-CoV, or influenza virus for 1 hour at 37°C, and then blocked with 5 wt.% BSA. SARS-CoV-2 NP scFv-Fc antibodies were added to the blocked well, and the sample was incubated for 1 hour at room temperature. After washing with TBST (Tris-buffered saline, 0.1 wt.% Tween 20), HRP-conjugated anti-human IgG antibody (#32935, Cell Signaling Technology, Danvers, MA) was added, and the sample was incubated for 1 hour at room temperature. After extensive washing with TBST, ELISA substrate (1-Step Ultra TMB-ELISA, Thermo Fisher

Scientific) was applied, and signals were obtained on a Synergy HTX plate reader (BioTek Instruments, Winooski, VT).

Procedure of biomarker sensing

We applied the prepared biomarker (e.g., IgG, SARS-CoV-2 NP antigen) solutions and cultured SARS-CoV-2 virus solutions of different concentrations to each one of the CNT sensor arrays where antibodies were bound respectively. The solutions of 10 μL were incubated on the CNT sensor array at room temperature for 15 min. After incubation, we changed the electrolyte in the PDMS well into 1 μM PBS solution and conducted the sensor measurement. Biomarker sensing was carried out by measuring the resistance of the CNT sensor by a digital multimeter (Fluke 83 V, Fluke Co., USA).

Supplementary Note 1. Machine Learning algorithms code

```
import pandas as pd
import matplotlib.pyplot as plt

from sklearn.metrics import mean_squared_error
from sklearn.metrics import r2_score
from sklearn.preprocessing import StandardScaler
from sklearn.model_selection import train_test_split
from sklearn.pipeline import Pipeline
from sklearn.model_selection import cross_val_score
from sklearn.model_selection import KFold
from sklearn.gaussian_process import GaussianProcessRegressor

### Load the data
df = pd.read_csv("your_experimental_data_file.csv")
predictors = df.iloc[:, 0:4]
response = df.iloc[:, 4]

# variable name
names = ('roughness', 'thickness', 'coverage', 'alignment')

### Machine-Learning Fit
# devide data to train set and test set
X_train, X_test, y_train, y_test = train_test_split(
        predictors, response, test_size=0.20, random_state=1, shuffle=True)

# Make train and test Dataframe
train = pd.DataFrame(X_train)
test = pd.DataFrame(X_test)

# Define a pipeline that standardize the data and the machine learning model
gpr = Pipeline([
        ('scaler', StandardScaler()),
        ('GPR', GaussianProcessRegressor(
))
])

# Fitting the data
gpr.fit(X_train, y_train)

### Analysis the Prediction
# get predicted result and save it to Dataframe
train['pred_gpr_train'] = gpr.predict(X_train)
test['pred_gpr_test'] = gpr.predict(X_test)
pred_gpr_train = train.iloc[:, 4]
pred_gpr_test = test.iloc[:, 4]

# Performance of prediction (RMSE, R square)
# 5-Fold Cross Validation check to prevent an overfitting
RMSE_test = mean_squared_error(y_test, pred_gpr_test)**0.5
RMSE_train = mean_squared_error(y_train, pred_gpr_train)**0.5

Kf = KFold(n_splits=5, shuffle=True, random_state=0)
cross_score_RMSE = cross_val_score(gpr, X_train, y_train,
scoring='neg_root_mean_squared_error', cv=Kf)
cv_RMSE=-cross_score_RMSE
cv_RMSE_mean = cv_RMSE.mean()
```

```
cross_score_R2 = cross_val_score(gpr, X_train, y_train, scoring='r2', cv=Kf)
cv_R2 = cross_score_R2
cv_R2_mean = cv_R2.mean()

R_2_test = r2_score(y_test, pred_gpr_test)
R_2_train = r2_score(y_train, pred_gpr_train)
print('RMSE_test', RMSE_test)
print('RMSE_train', RMSE_train)
print('R2_test', R_2_test)
print('R2_train', R_2_train)
print('cv_RMSE_mean', cv_RMSE_mean)
print (cv_RMSE)
print ('cv_R2_mean', cv_R2_mean)
print (cv_R2)

# Save 'true and predicted results' to .csv file
AP = pd.DataFrame()
AP.loc[:,'Sensitivity'] = response
AP.loc[:,'y_train'] = y_train
AP.loc[:,'y_trainP'] = pred_gpr_train
AP.loc[:,'y_test'] = y_test
AP.loc[:,'y_testP'] = pred_gpr_test
AP.to_csv("[Bio54_new]actual_predict_sensitivity_GPR.csv")

# Plot true and predicted results graph
fig, ax1 = plt.subplots()
ax1.scatter(y_train, pred_gpr_train)
ax1.scatter(y_test, pred_gpr_test, marker = 'x')
ax1.plot([-0.05, 0.15], [-0.05, 0.15], ls="--", c=".3")
ax1.set_ylabel('Predicted Sensitivity')
ax1.set_xlabel('Measured Sensitivity')
plt.tight_layout()

plt.show()
```

Supplementary Note 2. Nucleocapsid protein concentration from LoD in Fig.6c

In the detection of cultured SARS-CoV-2 virus in Figure 6c (with an LoD of 120 viruses/mL), the nucleocapsid proteins (NPs) in the virus were captured by the SARS-CoV-2 NP scFv-Fc antibodies. In other words, the viruses were lysed, and the NPs in viruses were detected. A single virus is known to contain more than 1000 NPs.[4] Therefore, based on the detection of NP, the LoD should be recalculated as follows.

$$\frac{120\, copies\ (virus)}{mL} = \frac{120\ \times 10^3\ NP\ copies}{mL}$$

$$\frac{120 \times 10^3\ NP\ copies}{mL} \times \frac{1\ mol}{6.022 \times 10^{23}} \times \frac{10^3\ mL}{1L} = 2 \times 10^{-16} M = 0.2\ fM$$

$$0.2\ fM = \frac{2 \times 10^{-16}\ mol}{1L} \times \frac{1L}{10^3\ mL} \times \frac{45.6 \times 10^3 g}{1\ mol}\ = 9.12 \times 10^{-15} g/mL = 9.12\ fg/mL$$

Our attained LoD of 9.12 fg/mL strongly correlates to the LoD obtained in Figure 6b (5.62 fg/ml). The reason for the slightly large value in the case of Figure 6c is likely due to the impurities in the solution.

Supplementary Videos description

Video S1. Numerical simulation visualizing trajectories of particles passing through the flat blade

Video S2. Numerical simulation visualizing trajectories of particles passing through the 1:2 blade

Video S3. Numerical simulation visualizing trajectories of particles passing through the 1:1 blade

	Processing conditions			Thin-film property (Average)				Performance	
Process Number	Concentration (mg/mL)	Temperature (℃)	Speed (mm/s)	Surface roughness (nm)	Thickness (nm)	Surface coverage (%)	Alignment (I_0/I_{90})	Sensitivity 1	Sensitivity 2
1	0.5	55	0.1	1.39	4.52	75.04	0.52	-0.00659	-0.00611
2	0.5	70	0.1	1.67	8.77	79.69	1.54	0.00558	0.00351
3	0.5	85	0.1	2.46	8.90	86.61	3.31	0.02576	0.02382
4	0.5	55	0.3	2.14	6.19	55.13	2.91	0.03319	0.03141
5	0.5	70	0.3	1.19	6.06	78.09	2.06	0.0169	0.01704
6	0.5	85	0.3	3.44	7.24	79.17	0.77	0.01241	0.01141
7	0.5	55	0.5	1.37	5.07	41.18	0.99	0	0
8	0.5	70	0.5	1.44	5.57	50.43	1.49	-0.00686	-0.00467
9	0.5	85	0.5	1.18	6.04	66.97	0.83	0.03701	0.03701
10	0.75	55	0.1	2.77	12.86	64.77	1.84	0.0043	0.01096
11	0.75	70	0.1	1.02	9.72	85.14	0.91	0.00152	6.92E-04
12	0.75	85	0.1	1.44	10.64	87.09	1.60	0.00557	0.00378
13	0.75	55	0.3	0.83	8.85	57.86	1.67	0.06304	0.07017
14	0.75	70	0.3	2.07	6.29	81.36	0.83	0.00515	0.00455
15	0.75	85	0.3	1.64	9.51	83.34	1.70	-0.00417	-1.54E-03
16	0.75	55	0.5	0.89	7.07	55.75	1.74	0.01759	0.01334
17	0.75	70	0.5	0.86	6.13	59.19	2.01	0.05857	0.05258
18	0.75	85	0.5	1.48	8.97	72.21	1.94	0.01906	0.01291
19	1	55	0.1	1.54	6.44	84.24	1.49	0.08899	0.0869
20	1	70	0.1	1.49	6.39	83.66	3.62	0.04201	0.04468
21	1	85	0.1	3.18	12.21	93.22	3.22	0.00289	0.00701
22	1	55	0.3	1.67	3.63	79.84	1.78	0.0803	0.07337
23	1	70	0.3	1.00	4.34	89.90	2.93	0.09301	0.09292
24	1	85	0.3	2.17	5.60	90.21	1.69	0.00504	4.02E-03
25	1	55	0.5	1.36	3.20	68.58	3.80	0.0623	0.06955
26	1	70	0.5	1.32	3.86	88.55	0.62	0.02501	0.02154
27	1	85	0.5	1.83	4.08	76.22	1.68	-0.0038	-0.00272

Table S1. Designed processing conditions of solution shearing, and resultant CNT thin-film properties (average values). Sensor sensitivities are highly dependent on the thin-film properties, needing to be optimized for accurate diagnosis.

Machine-learning techniques	Cross validated R^2 mean	Cross validation RMSE mean	R^2 on test set	RMSE on test set
Linear regression (LR)	0.1471	0.0273	-0.6168	0.0198
K-Nearest Neighbors (KNN)	0.2286	0.0252	-1.1334	0.0228
Gaussian process regression (GPR)	0.9248	0.0061	0.8294	0.0064
Random Forest (RF)	0.9543	0.0058	0.8393	0.0062
Stochastic Gradient Descent Regression (SGDR)	0.0350	0.0289	0.4920	0.0111

Table S2. Prediction performance of machine learning models to optimize the sensitivity of resistive immunosensors. (LR, Linear regression; KNN, K-Nearest Neighbors; GPR, Gaussian Process Regression; RF, Random Forest; SGDR, Stochastic Gradient Descent Regression). Root mean square error (RMSE) and coefficient of determination (R^2) were calculated using cross-validated (*k*=5) training set and test set.

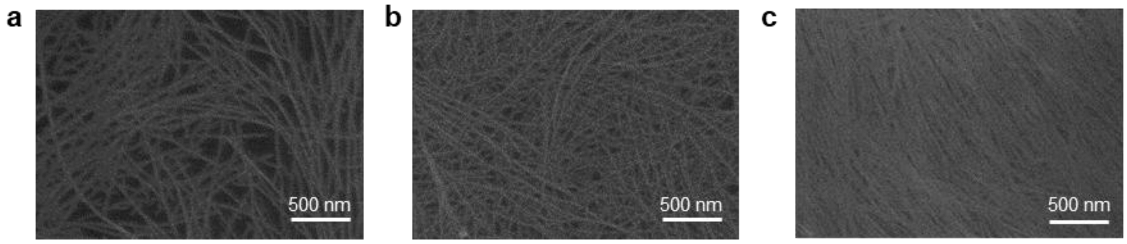

Figure S1. a-c) SEM images of CNT films, which shows the difference in film properties by changing the coating blade (flat blade (a), microstructured blade with 1:2 spacing (b), and 1:1 spacing (c)).

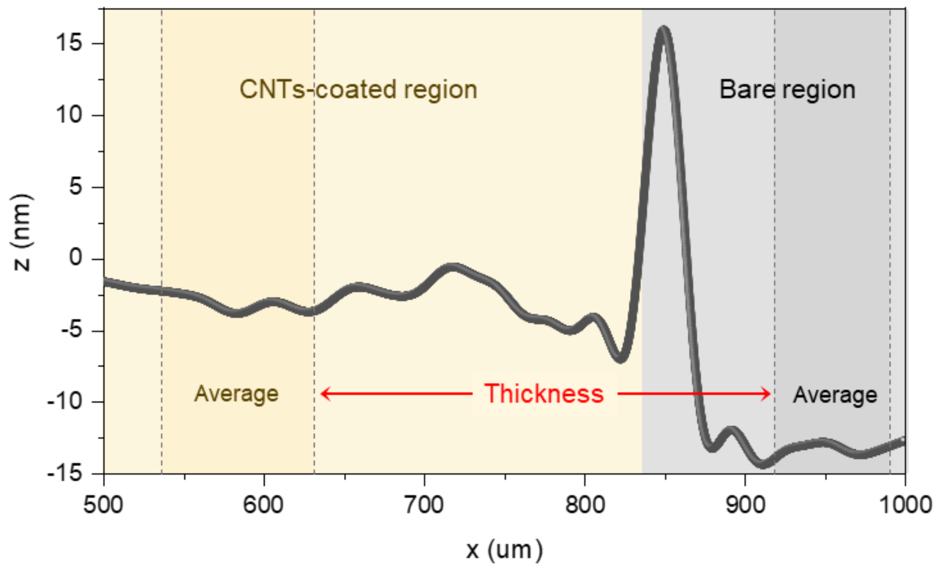

Figure S2. The analysis of calculating the thickness of CNT films coated with different processing conditions by measuring the difference between the average height value of CNTs-coated region and bare region through Alpha-Step (Alpha-Step D500, KLA Co., USA).

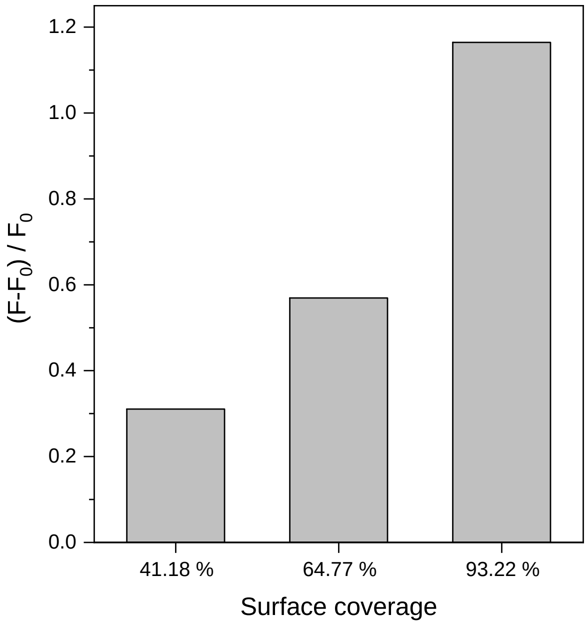

Figure S3. Fluorescence intensity of fluorescein isothiocyanate (FITC)-labeled anti-IgG immobilized on CNT films with 3 different surface coverage values. The fluorescence was measured at 525 nm (λ_{ex}= 490 nm) with 2 independent experiments for data reproducibility. The difference in fluorescence intensity testified to the surface coverage values calculated by the image processing program. (41.18 %: 0.31, 64.77 %: 0.57, 93.22 %: 1.16)

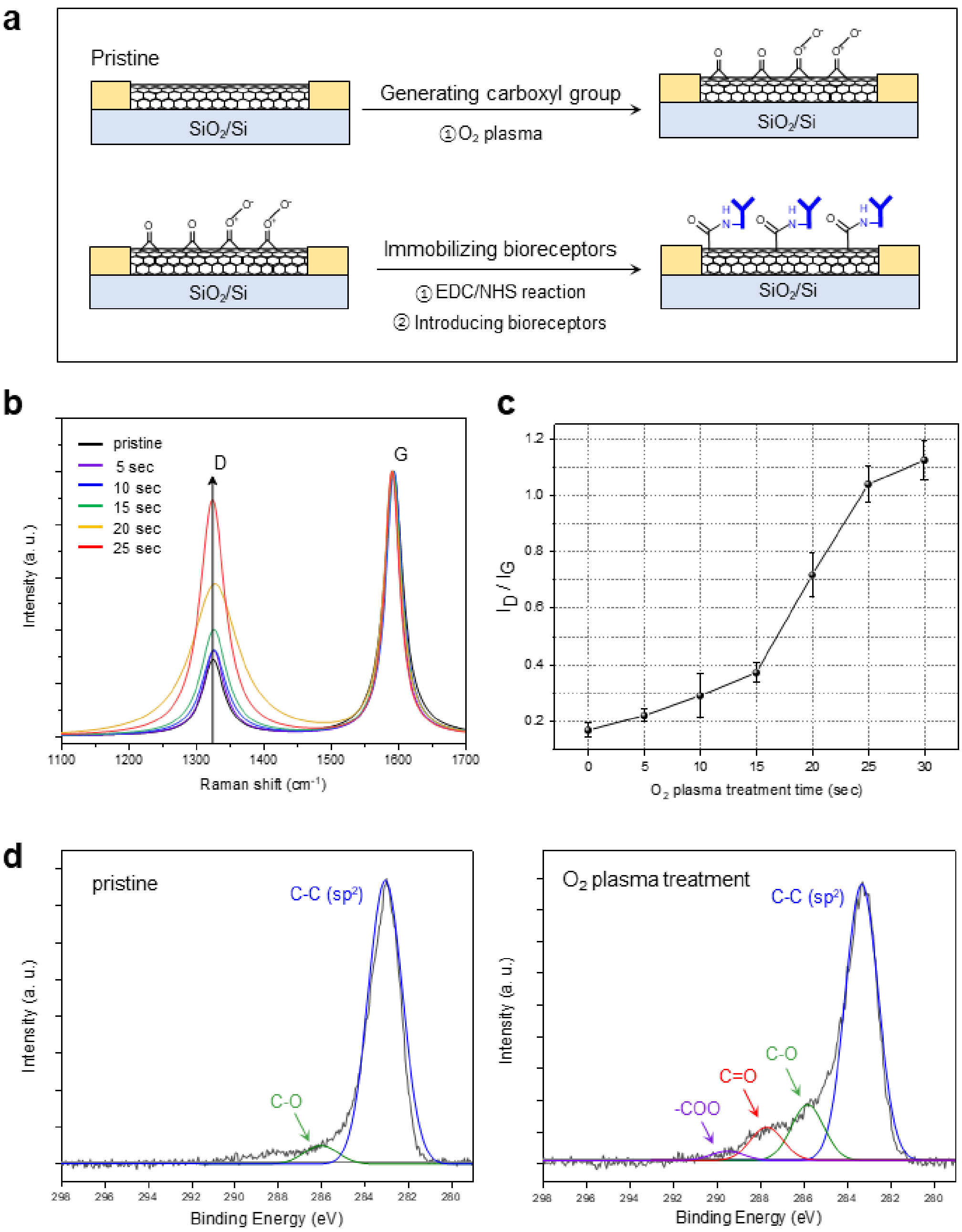

Figure S4. Functionalization of CNT films to immobilize bioreceptors. a) Schematic illustration of the functionalization procedure of CNT films with bioreceptors by EDC/NHS reaction. b) Change in the Raman spectra of the CNT films while increasing the degree of oxidation by O_2 plasma treatment. Raman spectroscopy showed the two characteristic peaks, D and G peak at 1325 and 1595 cm^{-1}. c) Changes in the ratio of intensity in D and G peak (I_D/I_G) from CNT films during the O_2 plasma-induced oxidation. The values of I_D/I_G gradually increased as the treating time increased. All the values represent the mean ± standard deviation (n=3). d) X-ray photoelectron spectroscopic (XPS) spectra of the CNT films before and after O_2 plasma treatment. After treatment, the content of oxygen molecules increased from 6.52 % to 23.8 %.

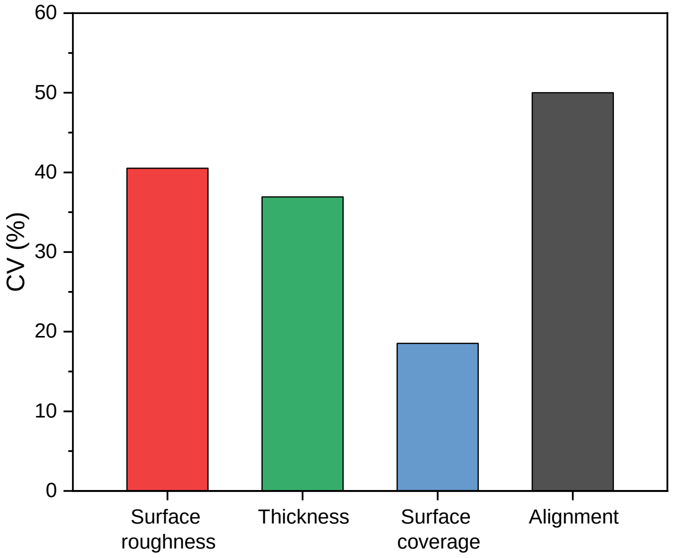

Figure S5. Comparison of coefficient of variation (CV) of the thin-films properties coated by the 1:1 spacing microstructured blade. (Surface roughness: 41.49 %, Thickness: 36.97 %, Surface coverage: 18.93 %, Alignment: 51.63 %)

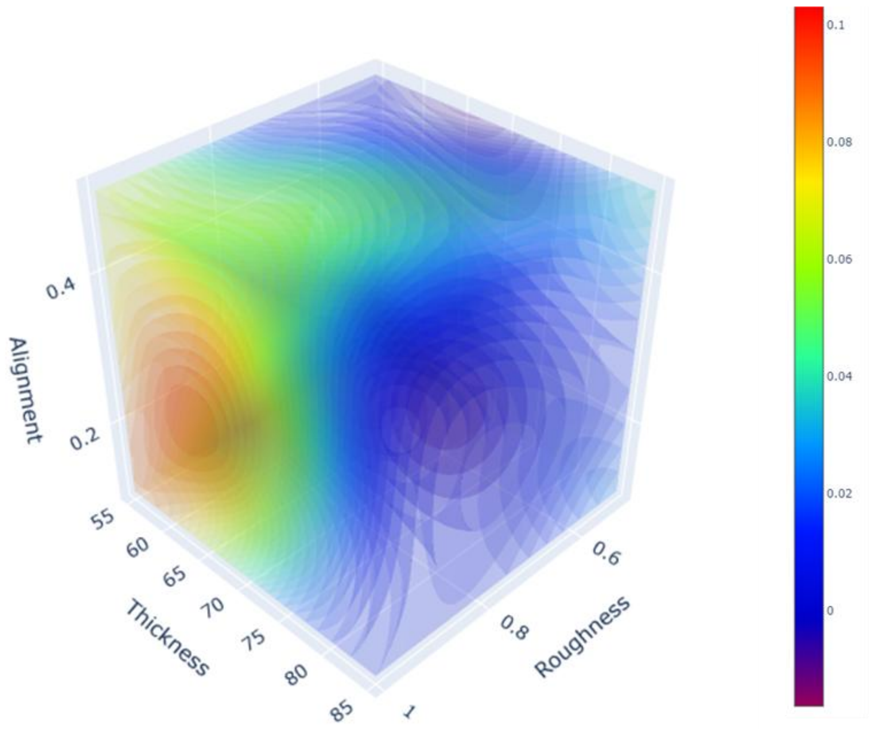

Figure S6. 3-dimensional map with 3 major film properties (i.e., surface roughness (*x*-axis), thickness (*y*-axis), and alignment (*z*-axis)), which shows the contribution of film properties to the sensitivity.

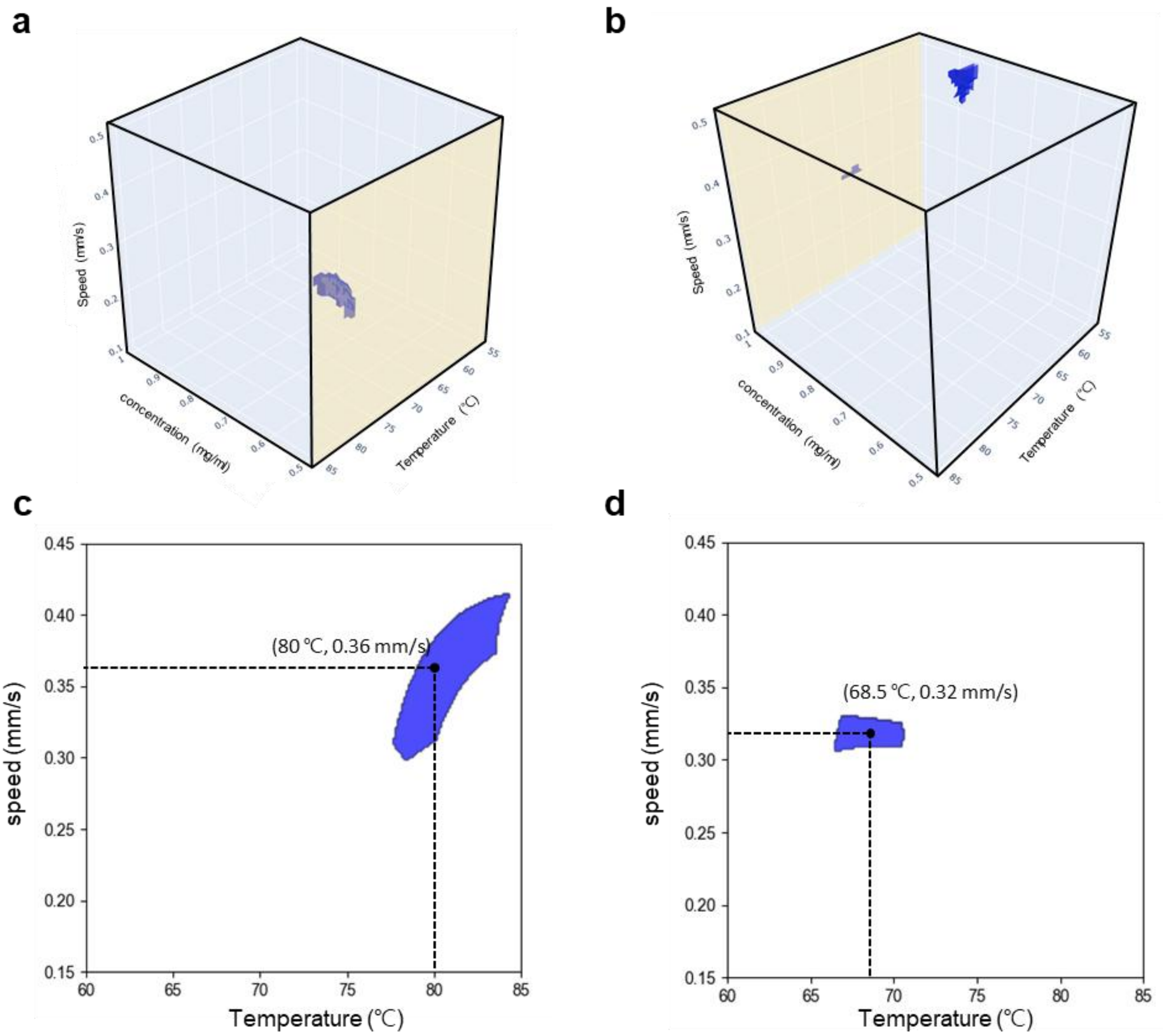

Figure S7. a,b) 3-dimensional cuboid parameter space, which shows the overlapped region satisfying the 'Low Alignment High Roughness;LAHR' (a) and 'High Alignment Low Roughness;HALR' (b) described in Figure 4c. c,d) By slicing the 3D parameter space with the concentration, range of temperature and speed for corresponding film properties can be achieved.

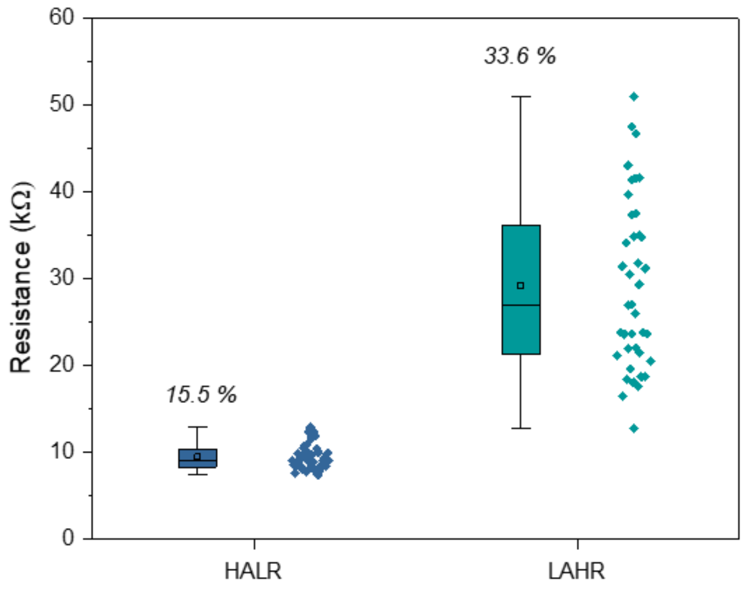

Figure S8. Comparison of HALR- and LAHR-based sensor array's resistance. Coefficient of variation (CV) values *(italicized)* were 2.2 times lower in HALR-based sensor array than that of LAHR-based sensor array.

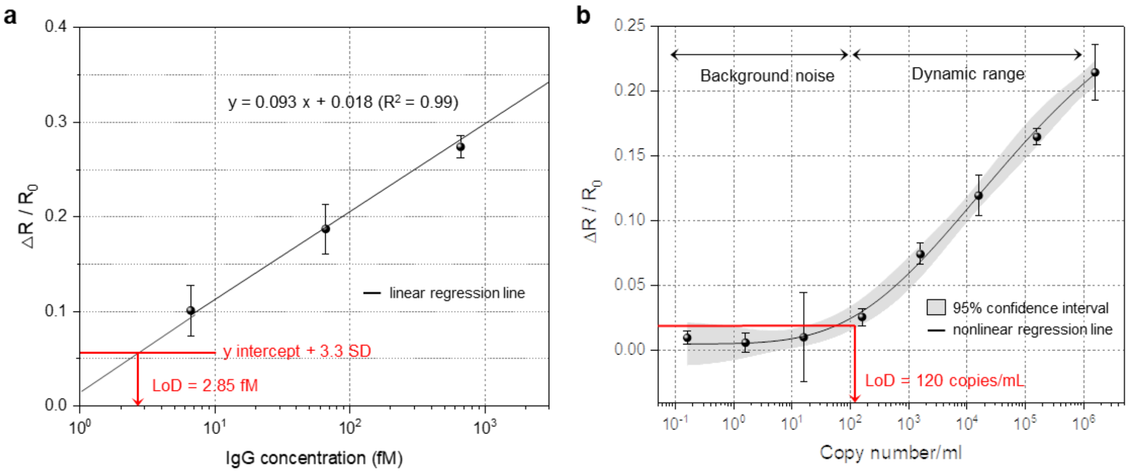

Figure S9. The analysis of LoD based on the linear and nonlinear regression line. a) In the case of linear regression, the LoD value is analyzed by following equation: $3.3 \times \sigma/S$, where σ is the residual standard deviation (SD) of the linear regression and S is the slope of the linear regression line. Here, the statistical confidence level is set as 3.3. The intersection line (red line) follows this equation. b) In the case of nonlinear regression, the LoD is defined as the concentration at the intersection (red line) between the upper bound of the 95% one-sided confidence interval of the background noise and the lower bound of the 95% one-sided confidence interval of the nonlinear regression line.[5]

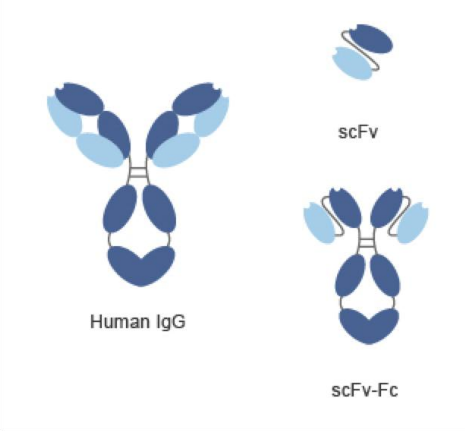

Figure S10. Schematic illustration of structures of recombinant antibody, including human IgG, SARS-CoV-2 NP-specific single-chain variable fragment (scFv), and single-chain variable fragment-crystallizable (scFv-Fc).

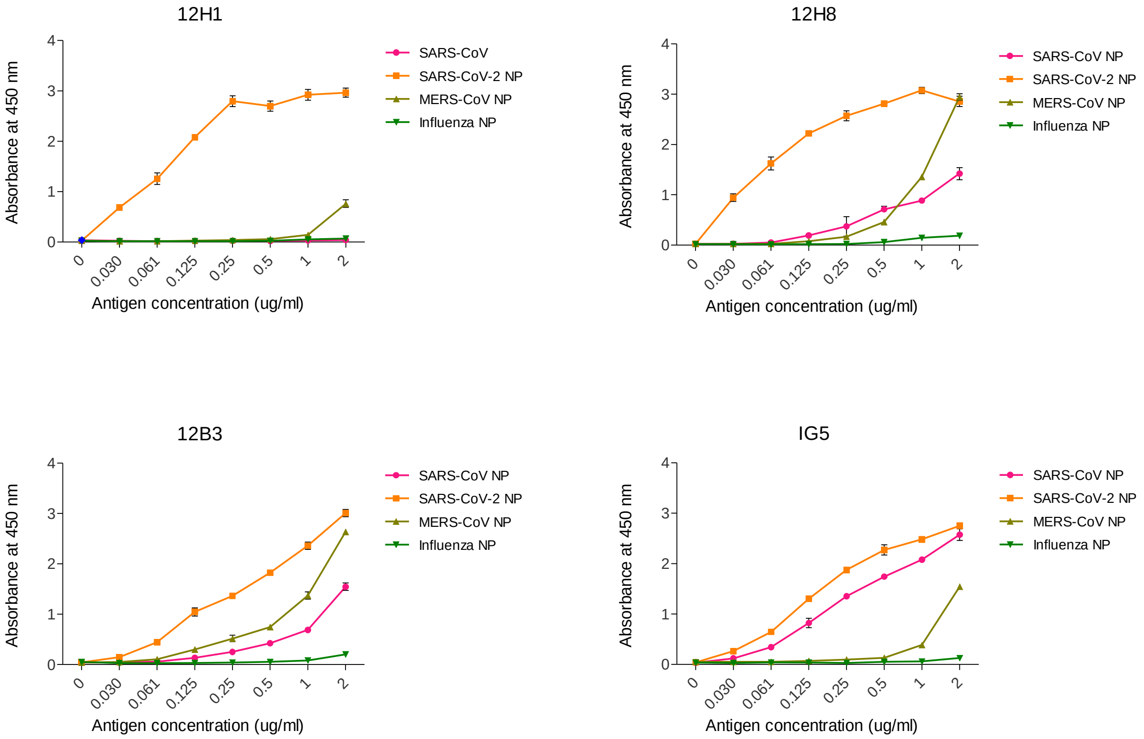

Figure S11. ELISA results for interaction between the scFv-Fc antibodies and NP antigens of the indicated virus. 12H1 scFv-Fc antibody showed the best sensitivity and selectivity.

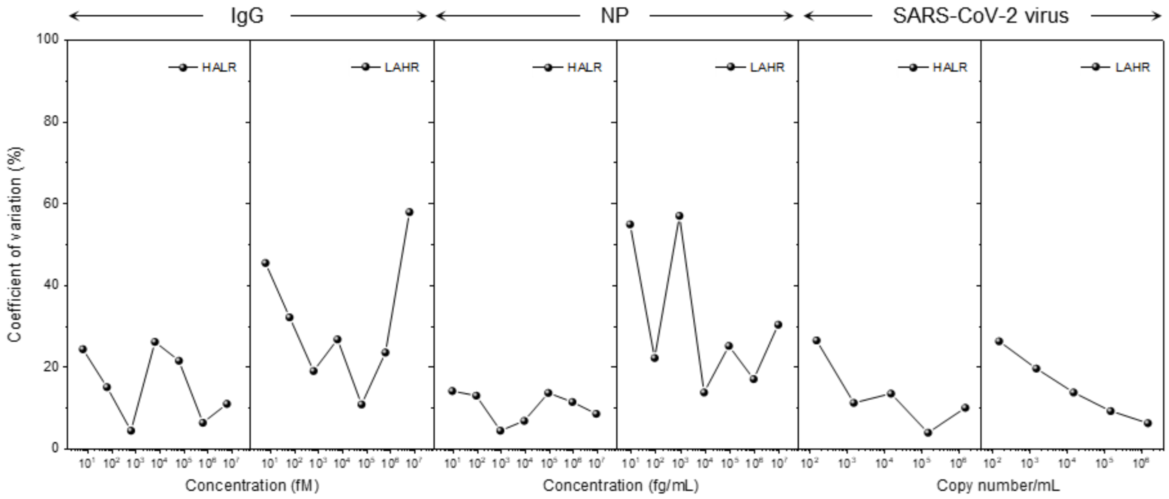

Figure S12. Coefficient of variation (CV) for the relative resistance changes of the HALR- and LAHR-based sensor array upon the exposure to IgG, nucleocapsid protein (NP) in buffer condition, and nucleocapsid protein in lysed SARS-CoV-2 virus.

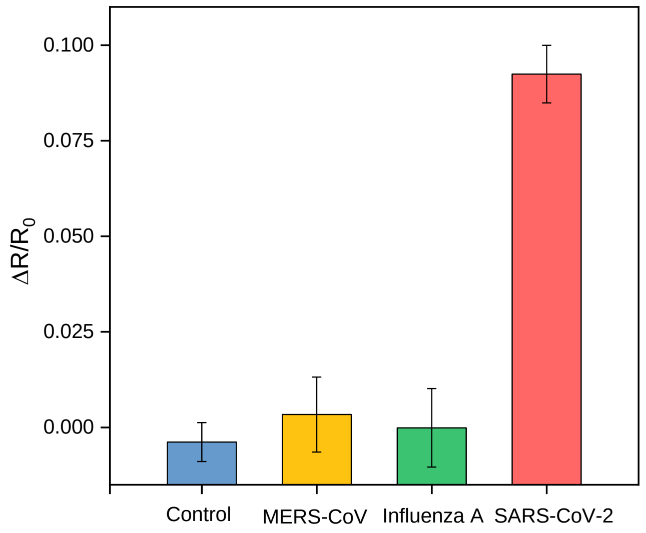

Figure S13. Selectivity of the HALR-based sensor towards various nucleocapsid proteins of MERS-CoV, Influenza A, and SARS-CoV-2 virus. 12H1 scFv-Fc antibody is used as the receptor, and the concentrations of nucleocapsid proteins are all 10 pg/mL. For each data, a different set of 3 sensors was used.

References

[1] J.-O. Kim, J.-C. Lee, M.-J. Kim, H. Noh, H.-I. Yeom, J. B. Ko, T. H. Lee, S.-H. Ko Park, D.-P. Kim, S. Park, *Adv. Mater.* **2018**, 30, 1800647.

[2] a) J.-C. Lee, M. Lee, H.-J. Lee, K. Ahn, J. Nam, S. Park, *Adv. Mater.* **2020**, 32, 2004864; b) M. Behr, T. Tezduyar, *Comput. Methods Appl. Mech. Eng.* **1999**, 174, 261.

[3] H. Y. Kim, J. H. Lee, M. J. Kim, S. C. Park, M. Choi, W. Lee, K. B. Ku, B. T. Kim, E. Changkyun Park, H. G. Kim, S. I. Kim, *Biosens. Bioelectron.* **2021**, 175, 112868.

[4] Y. M. Bar-On, A. Flamholz, R. Phillips, R. Milo, *eLife* **2020**, 9, e57309.

[5] a) G. Gauglitz, *Anal. Bioanal. Chem.* **2018**, 410, 5; b) C. Galitzine, J. D. Egertson, S. Abbatiello, C. M. Henderson, L. K. Pino, M. MacCoss, A. N. Hoofnagle, O. Vitek, *Mol. Cell. Proteomics* **2018**, 17, 913.
